# Serum ferritin and neutrophil-to-lymphocyte ratio predict all-cause mortality in patients receiving maintenance hemodialysis: a prospective study

**DOI:** 10.3389/fmolb.2024.1366753

**Published:** 2024-02-29

**Authors:** Jiamin He, Changyan Li, Jie Ge, Zhen Li, Lingyan Cao, Wenxing Fan, Yunzhu Peng, Qiongfang Li

**Affiliations:** ^1^ Department of Nephrology, The First Affiliated Hospital of Kunming Medical University, Kunming, Yunnan, China; ^2^ Organ Transplantation Center, The First Affiliated Hospital of Kunming Medical University, Kunming, Yunnan, China; ^3^ Department of Cardiology, The First Affiliated Hospital of Kunming Medical University, Kunming, Yunnan, China; ^4^ Department of Imaging, The First Affiliated Hospital of Kunming Medical University, Kunming, Yunnan, China

**Keywords:** ferritin, neutrophil-to-lymphocyte ratio, maintenance hemodialysis, all-cause mortality, predictors

## Abstract

**Introduction:** Maintenance hemodialysis is an effective treatment for end-stage renal disease patients. A critical factor contributing to the deterioration and death of maintenance hemodialysis patients is inflammation. Therefore, we focused on two inflammatory markers, serum ferritin and neutrophil-to-lymphocyte ratio, to speculate whether they could predict the prognosis of maintenance hemodialysis patients.

**Patients and methods:** We followed 168 patients with maintenance hemodialysis from July 2019 to July 2022 with the endpoint of all-cause death or follow-up completion. Receiver operating characteristic curves were plotted to assess the values of serum ferritin, neutrophil-to-lymphocyte ratio and serum ferritin combined with neutrophil-to-lymphocyte ratio to predict the outcomes of maintenance hemodialysis patients. Kaplan-Meier survival curves were constructed to compare survival rates over time.

**Results:** Receiver operating characteristic curves demonstrated that the best cut-off value of serum ferritin for predicting the prognosis of maintenance hemodialysis patients was 346.05 μg/L, and that of neutrophil-to-lymphocyte ratio was 3.225. Furthermore, a combination of both had a more excellent predicting value than either index (*p* < 0.05). Kaplan-Meier survival curve analyses revealed that low serum ferritin levels and low neutrophil-to-lymphocyte ratio had a higher probability of survival than high ferritin levels and high neutrophil-to-lymphocyte ratio, separately.

**Conclusion:** Elevated serum ferritin and neutrophil-to-lymphocyte ratio are closely related to all-cause mortality among maintenance hemodialysis patients, for which they may be predictors of all-cause mortality. Additionally, the combination of the two has a much higher predictor value for the prognosis of maintenance hemodialysis patients.

## 1 Introduction

Chronic kidney disease (CKD) has been world-recognized as a serious public issue, while the current number of CKD patients around the world has exceeded 700 million and is continually growing (GBD Chronic Kidney Disease Collaboration, 2020). As the renal function further deteriorates, by the time the condition progresses to end-stage renal disease (ESRD), patients have no choice but to choose renal replacement therapy such as hemodialysis, peritoneal dialysis, and renal transplantation, of which most ESRD patients receive hemodialysis (Zhang et al., 2020). Although its methods have been increasingly improved recently, there has been no significant reduction in patient mortality (Johansen et al., 2021). It has been demonstrated that the primary cause of death in maintenance hemodialysis (MHD) patients is cardiovascular events (Ahmadmehrabi and Tang, 2018). And inflammation is a key element in disease progression, all-cause and cardiovascular mortality in MHD patients (Liakopoulos et al., 2017; Cobo et al., 2018). Therefore, it is indispensable to recognize and intervene in the inflammatory status of MHD patients timely to promote better survival. However, traditional inflammatory markers, such as tumor necrosis factor-α and interleukin-6, are hardly tested routinely for the inconvenience and high cost of testing.

Ferritin is a primary intracellular iron storing protein and a vital inflammatory response protein in the body, closely related to iron metabolism and inflammatory response of the organism (Moreira et al., 2020). The latest view argues that ferritin is also an aggravating factor of inflammation (Ruscitti and Giacomelli, 2020). In ESRD patients receiving MHD, serum ferritin levels may often be elevated in varying amounts due to underlying systemic inflammation, regardless of the iron stores of the body (Kim et al., 2017). Previous studies have demonstrated that a high serum ferritin level is a significant predictor of death in MHD patients (Jenq et al., 2009; Kuragano et al., 2014; Kuragano et al., 2020). Neutrophil-to-lymphocyte ratio (NLR) is a novel inflammatory index proposed recently, reflecting the balance between anti-inflammatory and pro-inflammatory mechanisms of the body (Buonacera et al., 2022). It is also a prognostic indicator for morbidity and mortality of many diseases, including tumors, cardiovascular disease (CVD), liver disease, and surgical disease (Pineault et al., 2017). Studies have indicated that NLR can be used to predict survival rates in MHD patients (Zhang et al., 2021; Mayne et al., 2022). Serum ferritin and NLR are both easily accessible and inexpensive indicators in clinical practice. However, there is a lack of studies that consider both together. Accordingly, our study intended to assess the relations between serum ferritin and NLR with all-cause mortality in MHD patients and explore the combined predictive value of the two for patient prognosis.

## 2 Materials and methods

### 2.1 Patients

This prospective study recruited patients receiving regular hemodialysis at the First Affiliated Hospital of Kunming Medical University from July 2019 to July 2022. The number of cases in the hospital during the study period determined the sample size. Participants were selected based on the following criteria: Age ≥18 years; dialysis duration ≥3 months; dialysis 2–3 times a week for 4 h per session, with a blood flow of 200–300 mL/min; all treatment and management were performed following the Kidney Disease: Improving Global Outcomes (KDIGO) (Kidney Disease Improving Global Outcomes, 2012). Exclusion criteria included dialysis patients for acute kidney injury; patients with severe cardiovascular accidents lately; patients with acute-phase infections; those with active malignancy; those with liver disorders; those with hematological illnesses; those with recent surgery and trauma; those who transferred to kidney transplantation or peritoneal dialysis during follow-up; those with missing data; and those missing follow-up.

### 2.2 Follow-up

The follow-up of all patients began when patients were enrolled and started MHD treatment, with a final follow-up date of October 2022. And the follow-up endpoint is all-cause mortality or completion of follow-up.

### 2.3 Clinical and laboratory data collection

Participants performed blood tests every 3 months to determine their physical condition. The patient’s baseline clinical information was obtained at the time of enrollment, including age, gender, dialysis duration, primary illness, comorbid condition, and frequency of hemodiafiltration. And laboratory parameters including serum potassium (mmol/L), serum calcium (mmol/L), alkaline phosphatase (IU/L), serum albumin (g/L), triglycerides (mmol/L), total cholesterol (mmol/L), LDL cholesterol (mmol/L), HDL cholesterol (mmol/L), serum iron (μmol/L), total iron binding capacity (μmol/L), un-iron binding capacity (μmol/L), serum ferritin (μg/L), transferrin (g/L), urea (mmol/L), serum creatinine (μmol/L), uric acid (μmol/L), hemoglobin (g/L), absolute neutrophil value (109/L), absolute lymphocyte value (109/L), absolute monocyte value (109/L), platelets (109/L) and parathyroid hormone (pg/mL) were collected from every blood tests. Calculation of the ratio of absolute neutrophil value to absolute lymphocyte value resulted in NLR. Calculate the value of Kt/V, and Kt/V ≥ 1.2 was considered adequate for dialysis. Blood was not collected at the time of hemodialysis and 1 day after dialysis to avoid the effect of hemodialysis on the laboratory data. All laboratory data were carried out by our hospital laboratory with standardized and automated measures.

### 2.4 Statistical analysis

Statistical software SPSS 26.0 (IBM, Armonk, NY, United States) and MedCalc 19.0.4 (MedCalc Software bvba, Ostend, Belgium) were used for data analysis. Quantitative variables with normal distribution were presented as mean ± standard deviation, while non-normally distributed quantitative variables were presented as median and interquartile range. Qualitative variables were represented as frequencies and percentages. The Chi-square test, the Fisher exact test, the T tests and the Mann-Whitney *U* test were applied for between-group comparisons about baseline clinical information and laboratory parameters as appropriate.

The receiver operating characteristic (ROC) curves were built to estimate the values of serum ferritin, NLR and serum ferritin combined with NLR to predict MHD patients’ outcomes of death or survival, respectively. The diagnosis probability of serum ferritin combined with NLR was calculated by logistic regression. The area under the curve (AUC) was counted to quantify these potential survival bio-predictors and cross-compare their efficacy objectively. In addition, we calculated the specificity, sensitivity, and optimal cut-off values. Patients were separately divided into groups based on optimal cut-off values of serum ferritin and NLR. The patients with serum ferritin > the optimal cut-off value were grouped into the high-ferritin group, while those with serum ferritin ≤ the optimal cut-off value were grouped into the low-ferritin group. In the same way, patients were grouped into the high-NLR group and the low-NLR group. Kaplan-Meier survival curves revealed survival rates over time, while the Log-rank test was applied for further comparing comparison of two groups. Independent risk factors for death in patients with MHD were assessed by Cox proportional hazards regression (Forward: LR method). Univariate regression analysis was performed first, and the factors with *p* < 0.10 were incorporated for multivariate regression analysis. The difference in the analysis was statistically significant if *p* < 0.05.

## 3 Results

### 3.1 Baseline characteristics of the study patients

**FIGURE 1:**
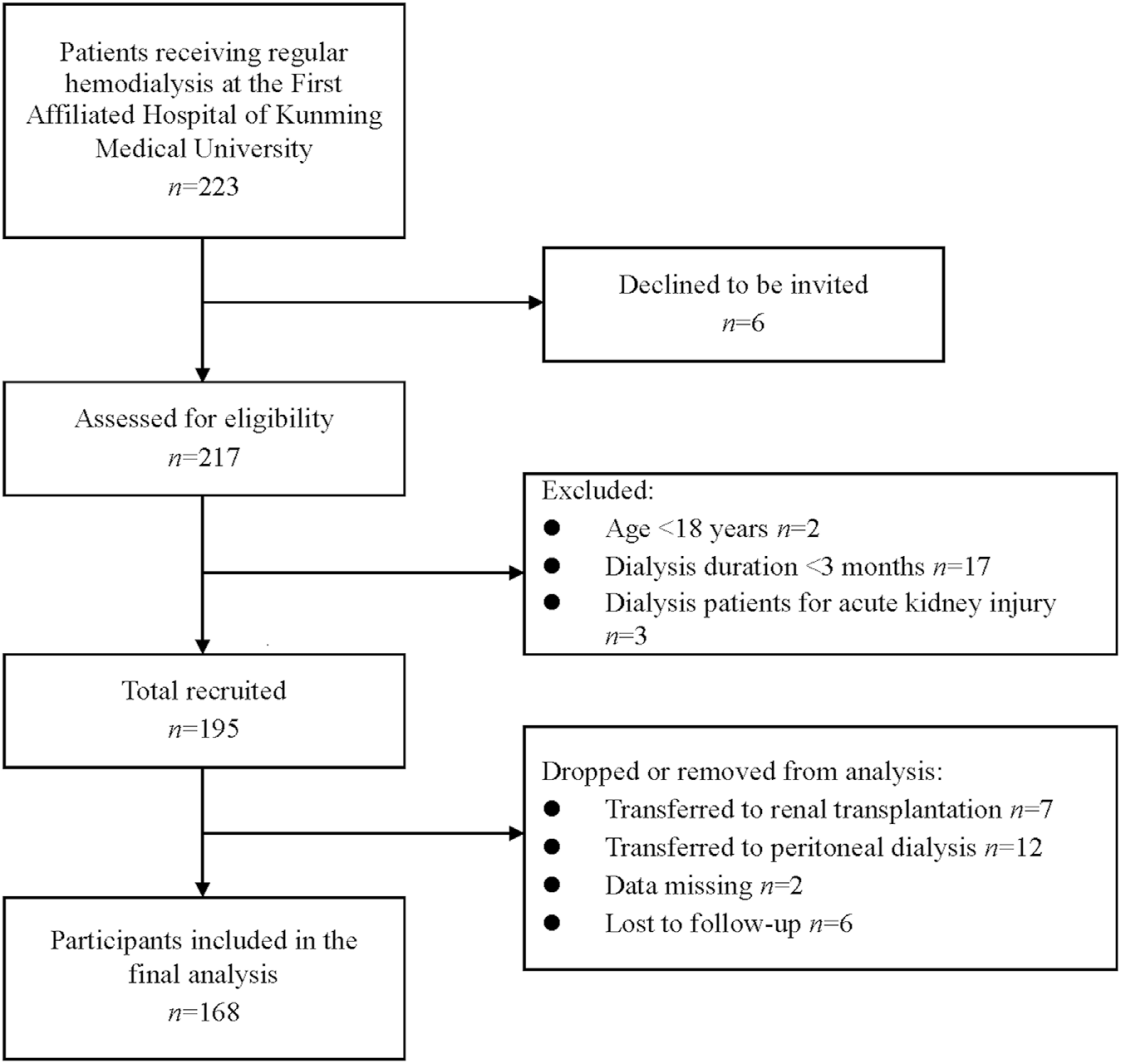
Flow chart of study enrollment.

Initially, 195 subjects were considered. During the follow-up period, 7 and 12 patients were transferred to renal transplantation and peritoneal dialysis, respectively, 2 patients had missing data, and 6 patients were lost to follow-up. Therefore, the final statistical analysis included 168 patients (Figure 1). Table 1 demonstrates the clinical and biochemical characteristics of the patients. The 168 MHD patients were primarily men (60.1%), with a mean age of 55.40 years and a median age on dialysis of 2.42 years. Chronic glomerulonephritis was the most common etiology, accounting for 42.3%, followed by diabetic nephropathy (26.8%), hypertensive nephropathy (13.7%), and chronic urate nephropathy (5.9%). MHD caused by other renal diseases, such as renal cysts, hydronephrosis, and drug-related renal damage, was presented in 19 patients (11.3%). Furthermore, among these patients, comorbidities of hypertension, diabetes, and heart failure were found in 142 (84.5%), 45 (26.8%), and 56 (33.3%) patients, respectively. Adequate dialysis was performed in 124 patients (73.8%). The frequency of hemodiafiltration ≥1 time per month was observed in 32 patients (19.0%).

**TABLE 1:** Clinical and biochemical characteristics of the 168 MHD patients.

Variables	Total (*n* = 168)	Survivors (*n* = 137)	Non-survivors (*n* = 31)	*p*-Value
Age (years)	55.40 ± 15.13	53.96 ± 14.60	61.77 ± 16.05	0.009
Gender, *n* (%)				0.506
Male	101 (60.1)	84 (61.3)	17 (54.8)	
Female	67 (39.9)	53 (38.7)	14 (45.2)	
Dialysis age (years)	2.42 (1.16, 4.25)	2.61 (1.17, 4.34)	1.99 (0.85, 4.20)	0.401
Primary disease, *n* (%)				0.098
Chronic glomerulonephritis	72 (42.9)	59 (43.1)	13 (41.9)	
Hypertensive nephropathy	23 (13.7)	21 (15.3)	2 (6.5)	
Diabetic nephropathy	44 (26.2)	31 (22.6)	13 (41.9)	
Chronic urate nephropathy	10 (5.9)	8 (5.8)	2 (6.5)	
Other disease	19 (11.3)	18 (13.1)	1 (3.2)	
Comorbidities, *n* (%)				
Hypertension	142 (84.5)	115 (83.9)	27 (87.1)	0.870
Diabetes	44 (26.2)	31 (22.6)	13 (41.9)	0.027
Heart failure	56 (33.3)	40 (29.2)	16 (51.6)	0.017
Dialysis adequacy, n (%)				0.027
Adequate	124 (73.8)	106 (77.4)	18 (58.1)	
Inadequate	44 (26.2)	31 (22.6)	13 (41.9)	
Frequency of hemodiafiltration, n (%)				0.647
≥1 time/month	32 (19.0)	27 (19.7)	5 (16.1)	
0 time/month	136 (81.0)	110 (80.3)	26 (83.9)	
Laboratory indicators				
Serum potassium (mmol/L)	5.33 ± 0.95	5.35 ± 0.95	5.26 ± 0.96	0.655
Serum calcium (mmol/L)	2.40 ± 1.52	2.41 ± 1.68	2.35 ± 0.31	0.835
Alkaline phosphatase (IU/L)	117.72 ± 101.77	116.21 ± 106.62	124.39 ± 77.84	0.687
Serum albumin (g/L)	41.53 ± 5.46	41.82 ± 5.60	40.25 ± 4.67	0.151
Triglycerides (mmol/L)	2.16 ± 6.59	2.38 ± 7.28	1.18 ± 0.48	0.361
Total cholesterol (mmol/L)	3.65 ± 1.09	3.70 ± 1.12	3.43 ± 0.90	0.220
LDL cholesterol (mmol/L)	2.12 ± 0.84	2.16 ± 0.84	1.93 ± 0.84	0.185
HDL cholesterol (mmol/L)	1.06 ± 0.33	1.06 ± 0.34	1.07 ± 0.34	0.832
Serum iron (μmol/L)	11.62 ± 6.40	11.24 ± 6.16	13.27 ± 7.28	0.111
Total iron binding capacity (μmol/L)	50.57 ± 22.81	51.86 ± 24.43	44.88 ± 12.25	0.125
Un-iron binding capacity (μmol/L)	37.18 ± 15.43	38.32 ± 15.26	32.16 ± 15.42	0.045
Serum ferritin (μg/L)	228.85 ± 350.93	166.50 ± 274.73	503.32 ± 497.80	0.001
Transferrin (g/L)	2.46 ± 0.69	2.54 ± 0.67	2.12 ± 0.67	0.002
Urea (mmol/L)	32.36 ± 70.95	34.04 ± 78.46	24.97 ± 7.12	0.522
Serum creatinine (μmol/L)	991.03 ± 336.74	1,014.51 ± 341.74	887.27 ± 296.92	0.057
Uric acid (μmol/L)	478.49 ± 106.72	479.24 ± 109.81	475.18 ± 93.41	0.849
Hemoglobin (g/L)	104.04 ± 22.64	103.87 ± 21.90	104.77 ± 26.04	0.841
Absolute neutrophil value (10^9^/L)	4.51 ± 1.84	4.45 ± 1.82	4.74 ± 1.96	0.434
Absolute lymphocyte value (10^9^/L)	1.38 ± 0.56	1.45 ± 0.55	1.07 ± 0.47	0.001
Absolute monocyte value (10^9^/L)	0.47 ± 0.21	0.46 ± 0.20	0.49 ± 0.24	0.584
NLR	3.63 ± 1.80	3.33 ± 1.43	4.92 ± 2.58	<0.001
Platelets (10^9^/L)	198.03 ± 80.91	204.09 ± 81.58	171.26 ± 73.22	0.041
Parathyroid hormone (pg/mL)	458.37 ± 413.71	445.70 ± 414.44	514.35 ± 412.51	0.406

**Abbreviations:** NLR, neutrophil-to-lymphocyte ratio.

Over the follow-up time, 31 died, and 137 survived. Table 1 also displayed a clinical characteristics comparison of survivors and non-survivors. Non-survivors were older (*p* = 0.009) and had a higher proportion of combined diabetes (*p* = 0.027), heart failure (*p* = 0.017), and inadequate dialysis (*p* = 0.027) compared to the survivors. However, the two groups did not differ significantly regarding combined hypertension and frequency of hemodiafiltration (*p* > 0.05). Moreover, no remarkable differences in other baseline characteristics, including gender, dialysis age and primary disease (chronic glomerulonephritis, hypertensive nephropathy, diabetic nephropathy, chronic urate nephropathy and other diseases) were found between survivors and non-survivors (*p* > 0.05). Concerning laboratory indicators, non-survivors had lower values of un-iron binding capacity (*p* = 0.045), transferrin (*p* = 0.002), absolute lymphocyte value (*p* = 0.001), and platelets (*p* = 0.041), but higher values of serum ferritin (*p* = 0.001) and NLR (*p* < 0.001). No other laboratory indicators differed significantly. None of the 168 patients had missing relevant data.

### 3.2 The efficacy of serum ferritin, NLR and serum ferritin combined with NLR in predicting outcomes of MHD patients

**FIGURE 2:**
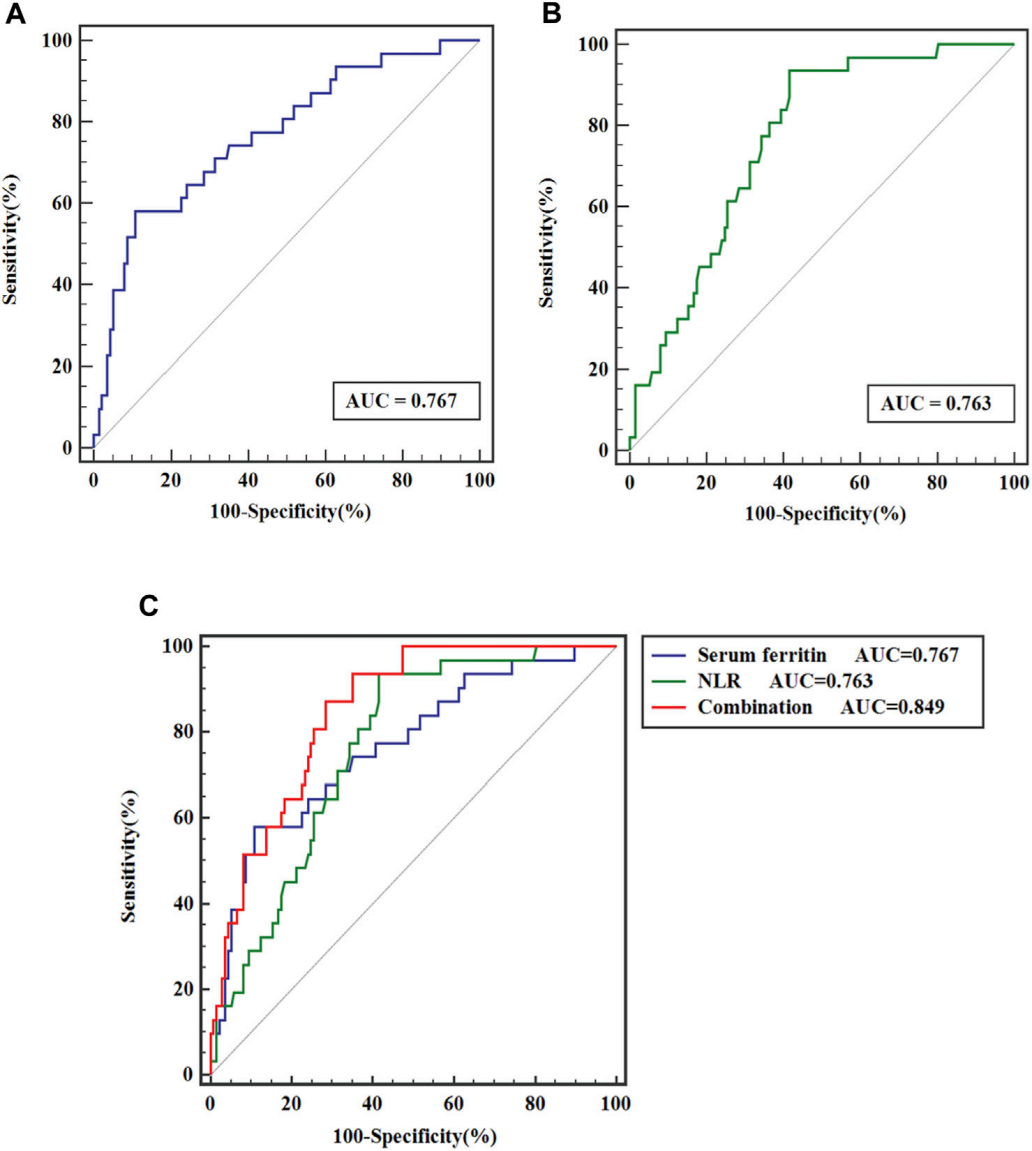
ROC curve analysis of **(A)** serum ferritin, **(B)** NLR and **(C)** serum ferritin combined with NLR for prediction of outcomes of MHD patients.

As depicted in Figure 2A, ROC curves showed that for predicting the prognosis of MHD patients, the AUC of serum ferritin was 0.767 (95%*CI*, 0.670–0.863; *p* < 0.001), with the best cut-off value of 346.05 μg/L, a sensitivity of 58.1%, and a specificity of 89.1%; and Figure 2B displays that the AUC of NLR was 0.763 (95%*CI*, 0.683–0.843; *p* < 0.001), with the best cut-off value of 3.225, a sensitivity of 93.5%, and a specificity of 41.6%. Moreover, as Figure 2C shows, the AUC of serum ferritin combined with NLR to distinguish death from survival in MHD patients was 0.849 (95%*CI*, 0.787–0.912; *p* < 0.001), greater than that of serum ferritin and NLR, with a statistically significant difference (*p* < 0.05).

### 3.3 Kaplan-Meier survival curves of MHD patients

**FIGURE 3:**
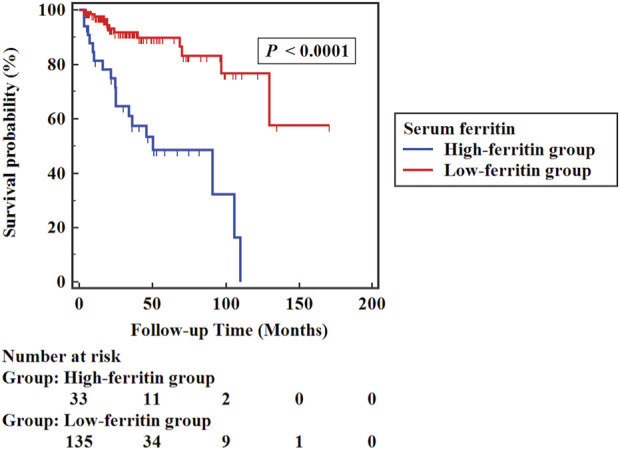
Kaplan-Meier survival curve of the high-ferritin group and the low-ferritin group.

The high-ferritin group (135 cases) was defined by serum ferritin >346.05 μg/L, and the low-ferritin group (33 cases) by serum ferritin ≤346.05 μg/L. All-cause mortality was markedly higher in the high-ferritin group (54.5%) than in the low-ferritin group (9.6%) (*p* < 0.05). As depicted in Figure 3, Kaplan-Meier survival curves indicated that low serum ferritin levels were correlated with a higher probability of survival significantly compared to high serum ferritin levels (Log-rank *χ* ^*2*^ = 28.709, *p* < 0.001).

**FIGURE 4:**
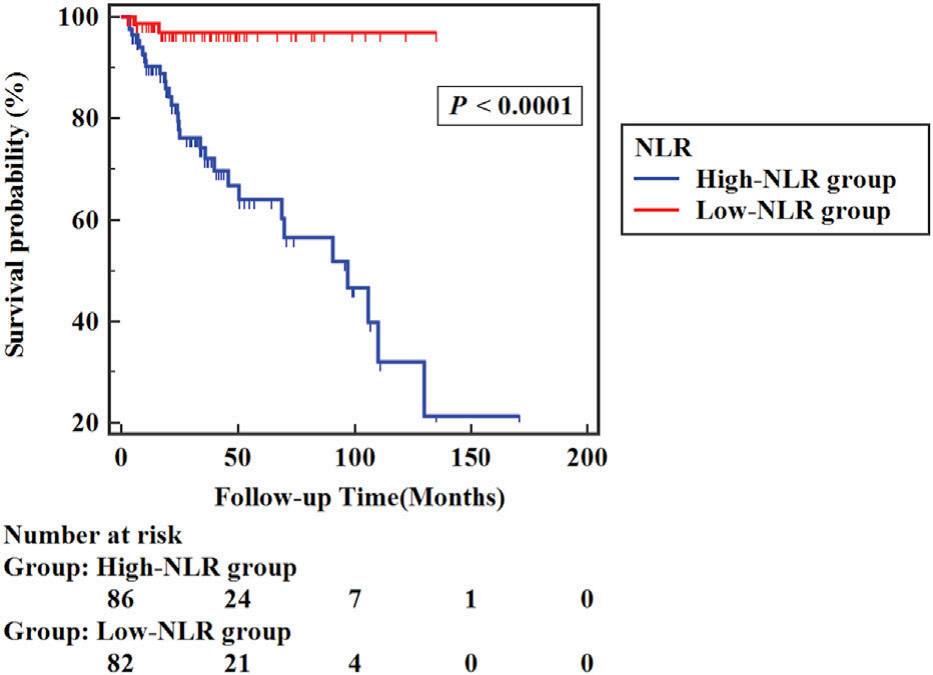
Kaplan-Meier survival curve of the high-NLR group and the low-NLR group.

Similarly, in the high-NLR group (86 cases), NLR >3.225, and in the low-NLR group (82 cases), NLR ≤3.225. The high-NLR group presented a considerably higher all-cause mortality rate (33.7%) than the low-NLR group (2.4%) (*p* < 0.05). Figure 4 shows the Kaplan–Meier survival curves between groups of the high-NLR group and the low-NLR group. Analysis of the results indicated that the probability of survival of patients was higher in the low NLR group (Log-rank *χ* ^*2*^ = 19.020, *p* < 0.001).

### 3.4 Independent risk factors for all-cause mortality in MHD patients

By the final follow-up visit, 31 patients had died. Table 2 indicates the Cox proportional hazard regression analysis of the variables related to all-cause mortality. Univariate regression analysis of each clinical and biological indexes revealed that variables with a *p*-value < 0.10 included older age (*p* = 0.012), comorbidities of diabetes (2.331 (1.125–4.828); *p* = 0.023) and heart failure (2.034 (0.993–4.163); *p* = 0.052), inadequate dialysis (2.017 (0.984–4.135); *p* = 0.055), lower serum albumin (*p* = 0.043), triglycerides (*p* = 0.029), total iron binding capacity (*p* = 0.047), un-iron binding capacity (*p* = 0.037), transferrin (*p* = 0.005), urea (*p* = 0.002) and serum creatinine (*p* = 0.003) levels, and higher serum iron (*p* = 0.048), serum ferritin (*p* < 0.001) and NLR (*p* = 0.001) levels. The above items were included in the multifactorial Cox regression analysis to adjust confounding variables. And the results showed that comorbid diabetes (2.243 (1.052–4.783); *p* = 0.036), comorbid heart failure (2.155 (1.021–4.547); *p* = 0.044), higher serum ferritin levels (*p* < 0.001), higher NLR levels (*p* = 0.004) and lower serum albumin levels (*p* = 0.019) were identified as independent risk factors for overall mortality in MHD patients. Absolute neutrophil and lymphocyte values were excluded from the Cox regression analysis, for they were part of NLR.

**TABLE 2:** A Cox proportional hazard regression analysis for the relevant factors of overall mortality in MHD patients.

Variables	Univariate		Multivariate	
	HR (95% *C.I.*)	*p*-Value	HR (95% *C.I.*)	*p*-Value
Age (years)	1.033 (1.007–1.060)	0.012		
Comorbidities				
Diabetes mellitus	2.331 (1.125–4.828)	0.023	2.243 (1.052–4.783)	0.036
Heart failure	2.034 (0.993–4.163)	0.052	2.155 (1.021–4.547)	0.044
Inadequate dialysis	2.017 (0.984–4.135)	0.055		
Laboratory indicators				
Serum albumin (g/L)	0.952 (0.908–0.998)	0.043	0.932 (0.879–0.988)	0.019
Triglycerides (mmol/L)	0.484 (0.252–0.929)	0.029		
Serum iron (μmol/L)	1.054 (1.001–1.111)	0.048		
Total iron binding capacity (μmol/L)	0.971 (0.944–1.000)	0.047		
Un-iron binding capacity (μmol/L)	0.957 (0.952–0.998)	0.037		
Serum ferritin (μg/L)	1.001 (1.001–1.002)	<0.001	1.001 (1.001–1.002)	<0.001
Transferrin (g/L)	0.446 (0.252–0.787)	0.005		
Urea (mmol/L)	0.923 (0.878–0.971)	0.002		
Serum creatinine (μmol/L)	0.998 (0.997–0.999)	0.003		
NLR	1.190 (1.074–1.318)	0.001	1.179 (1.053–1.319)	0.004

**Abbreviations:** NLR, neutrophil-to-lymphocyte ratio.

## 4 Discussion

The main findings presented in this study suggest that increased serum ferritin and NLR may be independent risk factors contributing to all-cause mortality for ESRD patients undergoing MHD and may be valuable predictors of outcomes for MHD patients. In addition, the combination of the two is more valuable in predicting the prognosis of patients receiving MHD than either index (*p* < 0.05).

Initially, ferritin was considered a reflection of iron storage status in the body and later a vital biomarker of inflammatory events in the body (Mahroum et al., 2022). Many inflammatory events of the organism, such as acute or chronic infections, liver disorders, hematological illnesses, and other malignant conditions, are always accompanied by an increase in serum ferritin level (Kernan and Carcillo, 2017). The latest ideas suggest that ferritin may have pathogenic effects and is not an innocent byproduct of inflammation (Sharif et al., 2018; Mahroum et al., 2022). High ferritin level is strongly associated with iron overload (Gattermann et al., 2021). In MHD patients, iron overload could promote the progression of kidney injury by increasing oxidative stress, stimulating inflammation, increasing tissue-iron deposition, and increasing the risk of infection in the organism (Batchelor et al., 2020). Meanwhile, iron overload has been demonstrated to contribute to an elevation in plasma heparin levels, which are strongly associated with the progress of CVD, such as atherosclerotic plaque formation, increased plaque instability and vascular sclerosis (Ueda and Takasawa, 2018). Unfortunately, CVD is widely known as the first death cause for MHD patients (Cozzolino et al., 2018). Our study found that the higher the serum ferritin levels in patients receiving MHD, the higher the risk of poor outcomes, which is consistent with previous findings (Karaboyas et al., 2018; Maeda et al., 2020). In addition, our study obtained the cut-off value of serum ferritin in predicting outcomes of MHD patients, namely, 346.05 μg/L. In an observational cohort study by Shoji et al., they discovered that serum ferritin was U-shaped correlated with overall mortality in MHD (Shoji et al., 2017). That appears a little different from the results of our study, which may be due to varying statistical methods and different patient baseline data. We analyzed it through ROC curves and Kaplan-Meier survival curves. However, Shoji et al. used a traditional baseline model and handled ferritin as a stratification factor (Gafter-Gvili et al., 2019). Furthermore, low serum ferritin levels imply iron deficiency anemia in the organism, which could increase the mortality risk of MHD patients (Afari and Bhat, 2016). Our study did not focus on the effect of low serum ferritin levels, which was a deficiency of this study.

Apart from serum ferritin, we also focused on the NLR--a novel proposed indicator of systemic inflammation in recent years. NLR was proved to have significant prognostic value among various diseases with inflammatory risk, such as CVD, COVID-19, renal cell carcinoma and sepsis (Afari and Bhat, 2016; Çalışkan et al., 2018; Huang et al., 2020; Pirsalehi et al., 2020). In addition, the research by Yilmaz et al. indicated that high NLR was associated markedly with the existence of uric acid and proteinuria in CKD patients (Yilmaz et al., 2017). Wongmahisorn et al.’s research revealed that high NLR was also remarkably correlated with early failure of hemodialysis arteriovenous fistulas in patients receiving MHD (Wongmahisorn, 2019). All those factors are detrimental to the prognosis of MHD patients. In our study, Survival rates were lower in those with higher NLR. Previous studies have reported similar results but did not get a clear threshold to suggest its optimal range (Ouellet et al., 2016; Zhang et al., 2021). Instead, we got the cut-off value for NLR in predicting the prognosis of MHD patients, which is 3.225.

Besides, we plotted the ROC curve for predicting patient outcomes by the combination of serum ferritin with NLR, which shows a more excellent predicting value, with an AUC of 0.849. That appears to be a novel finding, but there is still a need for more multi-center, large-sample clinical trials to further validate it. Therefore, clinicians should be more attentive to serum ferritin and NLR since they are both readily available and low-cost as vital predictors of overall mortality in MHD patients.

In addition, serum ferritin and NLR can serve as targets for therapeutic interventions, not just as biomarkers for disease prognosis. One intervention measure is to restrict the generation of inflammation molecules by avoiding various infections, congestive heart failure, dialyzer incompatibility, and poor water quality (Cobo et al., 2018). Furthermore, establishing a positive living style with appropriate physical exercise, a healthy diet and non-smoking should be significantly encouraged to decrease inflammation (Jankowska et al., 2017). Finally, MHD patients with anemia should take iron supplements appropriately to keep the serum ferritin within a reasonable range, improving their prognosis (Fishbane et al., 2014). However, there remains a lack of consensus regarding the maximum standard serum ferritin levels limit in MHD patients (Malaki, 2018). KDIGO recommends iron supplements if ferritin ≤500 μg/L for CKD patients with anemia (Kidney Disease Improving Global Outcomes, 2012). Practice guidelines from the Renal Association (2017) raise the upper limit of serum ferritin to 800 μg/L when taking iron supplements (Mikhail et al., 2017). The threshold value of serum ferritin derived in our study slightly differs from the above values, probably owing to the Chinese population studied and the small number of samples. Therefore, our findings are more applicable to the Chinese probably. Nevertheless, there is still a need for more high-quality clinical studies to explore this issue.

Meanwhile, the results of the Cox proportional hazard regression analysis showed that combined diabetes, combined heart failure and lower serum albumin levels were independent risk factors of all-cause death among MHD patients. A systematic evaluation noted that diabetes and cardiovascular disease can significantly increase the risk of cardiac death in MHD patients (Ma and Zhao, 2017). Serum albumin may reflect the nutritional status of the body. Previous research has demonstrated that hypoalbuminemia is a significant risk factor for all-cause mortality in MHD patients (Tang et al., 2021). So, it is essential to attend to the management of clinical comorbidities such as diabetes, heart failure and malnutrition to assess patient prognosis comprehensively.

There are still deficiencies in this study. Since patients’ treatment protocols were constantly adjusted according to the actual condition of the organism, some potentially confounding factors, such as medication status and hemodialysis mode, were not incorporated in this study, and their effects on the outcome cannot be excluded. Furthermore, as mentioned previously, the small number of samples may result in biased results. Therefore, there remains a need for clinical trials from multicenter with large samples to validate further the relevance of serum ferritin and NLR to the outcomes of MHD patients.

## 5 Conclusion

Serum ferritin and NLR can be predictive factors in the prognosis of MHD patients. Moreover, the combination of the two has a higher predictive value.

## Data Availability

The raw data supporting the conclusion of this article will be made available by the authors, without undue reservation.
